# Evaluation of the anti-inflammatory effect of ectoine–leflunomide combination in adjuvant-induced arthritis in rats, and its colonic protective action against leflunomide-induced gastrointestinal injury

**DOI:** 10.1007/s10787-026-02168-8

**Published:** 2026-03-23

**Authors:** Nour El-Hoda Gamal Mosaad, Eman Abdel-Fattah Selima, Rowaida Refaat Shehata, Amany Hussein Kazem, Mona Abdelrazek Salama, Ghada Osama Hammad

**Affiliations:** 1 Department of Pharmacology, Medical Research Institute, Alexandria University, Alexandria, Egypt; 2 Department of Pathology, Medical Research Institute, Alexandria University, Alexandria, Egypt; 3 Department of Pharmacology and Therapeutics, Faculty of Pharmacy, Pharos University in Alexandria, Alexandria, Egypt

**Keywords:** Ectoine, Leflunomide, Adjuvant arthritis, Combination therapy, Colitis

## Abstract

Rheumatoid arthritis (RA) is an autoimmune disease with an intriguing pathophysiological mechanism that primarily affects the joints and leads to extra-articular manifestations. Leflunomide is one of the most effective disease-modifying anti-rheumatic drugs (DMARDs), utilized in RA treatment, but its use comes with frequent gastrointestinal adverse effects, which might necessitate therapy discontinuation. The current work aimed to explore the antirheumatic and gastroprotective effects of ectoine, a compatible solute found in nature, on adjuvant-induced arthritis (AIA) in rats, and to compare its efficacy with that of leflunomide. In this study, animals were divided into five groups: Normal control rats, Untreated adjuvant arthritis (AA) rats, Leflunomide group treated with 10 mg/kg/day, Ectoine group treated with 100 mg/kg/ day, and AA rats treated orally with the combination of ectoine and leflunomide daily with the same dosses. Ectoine administration significantly abolished both articular and extraarticular inflammatory responses in AA rats. Serum levels of nitric oxide (NOx), Interleukin-1β (IL-1β), and anti-cyclic citrullinated peptide (anti-CCP) were significantly reduced, with a significant increase in IL-10. It dramatically suppressed tibiotarsal tissue expression of tumor necrosis factor-α (TNF-α), nuclear factor-kappa B-p65 (NF-κB-p65), receptor activator of NF-κB ligand (RANKL), and matrix metalloproteinase-9 (MMP-9). Colonic examination also showed lower expression levels of TNF-α, NF-κB-p65, intracellular adhesion molecule-1 (ICAM-1), and MMP-9, in addition to a significant antioxidant effect by enhancing reduced glutathione synthesis (GSH) and reduction of myeloperoxidase (MPO) and malondialdehyde (MDA) levels. Our results demonstrate that ectoine has a strong potential for being an efficient anti-inflammatory and anti-rheumatic agent.

## Introduction

Rheumatoid arthritis (RA) is one of the most prevalent systemic autoimmune disorders that causes synovial inflammation, articular damage, and bone erosion. It is not restricted to joints, but patients frequently suffer from multiple extra-articular manifestations (Sharma and Goel 2023). Inadequate treatment of RA can cause chronic pain, deformity, and permanent disability (Smolen et al. 2023). Unfortunately, it is an incurable disorder, and all therapeutic guidelines aim at slowing or halting inflammatory joint structure deterioration, pain alleviation, enhancing mobility, and improving patients’ quality of life (Chatterjee et al. 2024). Corticosteroids, non-steroidal anti-inflammatory medications (NSAIDs), disease-modifying anti-rheumatic drugs (DMARDs), either conventional synthetic (csDMARDs) targeted DMARDs, or the most recent class, biological DMARDs (bDMARDs), are among the several therapeutic modalities that have long been licensed for the treatment of RA (Bullock et al. 2019). Despite the proven efficacy of these lines of therapy, their use is associated with several limitations.

Many csDMARDs have been utilized for decades in RA management, with methotrexate being the anchor first-line agent in this class. Unfortunately, its use is often limited by hepatotoxicity, gastrointestinal disturbances, and haematological toxicities that are frequently encountered in patients (Wei et al. 2022). Leflunomide is one of the csDMARDs that acts by inhibiting mitochondrial enzyme dihydroorotate dehydrogenase (DHODH), which is essential for the de novo synthesis of pyrimidines, mainly uridine monophosphate (UMP). This mechanism causes a reduction in pyrimidine availability needed for the proliferation of activated T lymphocytes. Consequently, leflunomide prevents T-cell clonal expansion that dominates the autoimmune response in RA (Dhiman and Garkhal 2025). Leflunomide can inhibit different signalling pathways such as Janus kinase/signal transducers and activators of transcription (JAK/STAT), and NF-κB, with a subsequent decrease in pro-inflammatory cytokines, including IL-2, TNF-α, and interferon-gamma (IFN-ɣ). It also inhibits peripheral blood mononuclear cell (PBMC) activity, further dampening the immune response (Fragoso and Brooks 2015). It is widely prescribed to patients with an absolute contraindication to standard methotrexate therapy or in combination with it in refractory cases (Alfaro-Lara et al. 2019; Brown et al. 2016).

While being a highly effective therapeutic option for RA, leflunomide administration has been associated with frequent gastrointestinal side effects such as nausea, vomiting, diarrhea, and abdominal pain that may take place in up to 20% of patients (Smolen et al. 1999). Although rare, a serious and potentially life-threatening complication, leflunomide-induced colitis has been reported (Yang et al. 2024). To relieve the severe leflunomide-induced GIT problems, discontinuation of therapy is necessary, especially in cases of colitis (Kabir et al. 2024).

Despite the extensive research, the exact mechanism of leflunomide-induced gut inflammation is not yet understood; however, several mechanisms have been suggested. It has been proposed that leflunomide gastrointestinal side effects and the acute diarrhea frequently occurring may be a consequence of its immunomodulatory effect and its impact on activated T-lymphocytes (Chis et al. 2021). Another theory was attributing leflunomide’s toxicity to genetic differences associated with metabolism and clearance, of the drug and its active metabolite, teriflunomide (Duquette et al. 2016; Lebrun et al. 2018), It was suggested that poor metabolizers of leflunomide due to lower CYP2C19 activity would have lower concentrations of teriflunomide and, subsequently, lower intestinal toxicity (Grabar et al. 2009). However, future research should concentrate on metabolites mediating leflunomide toxicity.

A growing number of RA patients have started turning to natural products to alleviate symptoms of the disease and to avoid the severe side effects of conventional therapy. Ectoine is a naturally compatible solute protectant with a cyclic tetrahydro-pyrimidine structure. It is synthesized by halophilic bacteria under extreme environmental conditions, such as heat, drought, or high salinity (Zaccai et al. 2016). Ectoine can bind to water molecules to create a hydration shield that protects cellular biomolecules and proteins from damage and irreversible denaturation, in addition to acting as a cellular membrane stabilizer (Czech et al. 2018).

Based on its protective features, ectoine has been considered for the management of various inflammatory conditions. In acute bronchitis and acute respiratory infections, ectoine inhalation has demonstrated a significant prohibition of neutrophilic lung inflammation (Tran et al. 2019). In dermatological conditions such as atopic dermatitis, ectoine protects keratinocytes by reducing oxidative stress and their apoptosis while enhancing keratinocyte viability (Xu et al. 2025). In ocular inflammation, including dry eye syndrome, ectoine downregulates the expression of pro-inflammatory mediators: IL-1β, TNF-α (Chen et al. 2024), while also enhancing tear film stability and offering remarkable protection of the ocular surface (del Olmo et al. 2025). Emerging evidence also indicates that ectoine may benefit gastrointestinal inflammatory conditions such as irritable bowel disease (IBD) by inhibiting lymphocytic mucosal infiltration, reducing oxidative stress, and inhibiting TNF-α, intracellular adhesion molecule-1 (ICAM-1), and prostaglandin E_2_ synthesis (Bethlehem and Echten-Deckert 2021).

Based on promising evidence showing the anti-inflammatory potential of ectoine as a natural alternative or adjunctive therapy in immunological disorders, this study aimed to assess the potential anti-inflammatory and anti-rheumatic benefits of ectoine in rats with adjuvant-induced arthritis in comparison to one of the standard therapies, leflunomide, together with investigating the prospect of reversing the intestinal changes brought on by leflunomide when used in combination therapy with the osmoprotectant and membrane stabilizer ectoine.

## Materials and methods

### Animals

Forty adult male Sprague-Dawley rats, weighing 170–200 g, were obtained from the animal house of Alexandria University’s Medical Research Institute and were kept under observation with free access to food and water for one week before the study. All animal procedures were performed according to the regulations of the National Research Council’s Guide for the Care and Use of Laboratory Animals (Council et al. 2010), and the Egyptian guide for the care and use of laboratory animals recommendations (Fahmy and Gaafar 2016). Our study protocol was approved by the Ethics Committee of the Medical Research Institute, University of Alexandria (AlexU-IACUC-AU0122262222).

### Drugs & chemicals

Complete Freund’s adjuvant at a concentration of 10 mg/ml of heat-killed Mycobacterium tuberculosis was purchased from Difco Laboratories (Co, USA) and used for the induction of adjuvant-induced arthritis (AIA). The drugs used in the study were: Leflunomide (EVA Pharma-Egypt) and Ectoine (Sigma-Aldrich Chemical- Co.St. Louis, USA).

### Induction of Adjuvant-induced arthritis & experimental design

AIA model was induced in the experimental animals by injecting 0.1 ml of 10% w/v complete Freund’s adjuvant subcutaneously at the base of the tail. Chronic inflammation was allowed to progress for 14 days before commencing drug therapy,

Animals were allocated randomly into five groups, each consisting of eight rats:

- Group I: Normal control rats receiving 0.9% saline orally daily.
- Group II: Untreated adjuvant arthritis (AA) rats 0.9% saline orally daily.
- Groups III: AA rats treated orally with leflunomide dissolved in 0.9% saline, at a dose of 10 mg/kg/day (Gowayed et al. 2015).
- Group IV: AA rats treated orally with ectoine dissolved in 0.9% saline, at a dose of 100 mg/kg/ day (Abdel-Aziz et al. 2013).
- Group V: AA rats treated orally daily with the combination of both drugs with the same previously mentioned doses.

All drugs were administered daily for two weeks, from the 15^th^ day post-induction till the 28^th^ day of the experiment.

### Change in body weight

Animals’ weight was recorded on day 0 (before AA induction), then measured every other day till the day of the study. The percentage change in BW from day 0 to day 29 was calculated and used to evaluate the effect of chronic inflammation and treatments on body weight.

$$\begin{aligned} & {\mathrm{Percent}}\;{\mathrm{change}}\;{\mathrm{in}}\;{\mathrm{Body}}\;{\mathrm{weight}} \\ & \quad = ~\frac{{Body\;weight\;on\;day\;29 - Body\;weight\;on\;day\;0}}{{Body\;weight\;on\;day\;0}} \times 100 \\ \end{aligned}$$

### Spleen and liver mass indices

After collecting the blood, the spleens and livers of experimental animals were excised, washed, blotted dry, and weighed. The spleen and liver mass indices (ratio of spleen and liver weights in mg to animal weight in g) were employed as evidence of inflammation (Bendele 2001; Zhang et al. 2014).

### Assessment of arthritis progression

Hind paw swelling was assessed by measurement of the ankle diameter using a manual Vernier calliper in all experimental animals on day 0, 9, 14,18, 21, 25, and on the sacrifice day, the 29^th^ day.

### Serum inflammatory parameters

On day 29, rats were sacrificed by cervical dislocation. Blood samples were collected from the posterior vena cava and serum was separated and stored at − 80 °C for the determination of serum levels of anti-cyclic citrullinated peptide antibodies (Anti-CCP) (My Biosource^®^ USA), Interleukin 1-β (IL-1β) (My Biosource^®^ USA), IL-10 (Invitrogen TM, Thermo Fisher Scienific, USA), and tissue necrosis factor-α (TNF-α) (My Biosource^®^ USA) using enzyme-linked immunosorbent assay (ELISA) kits, following the manufacturer’s instructions. Serum Nitrate/nitrite (NOx) levels were evaluated via the Griess reaction (Miranda et al. 2001).

### Tibiotarsal tissue parameters

The subcutaneous tissue of the hind paw surrounding the tibiotarsal joints was excised, washed with ice-cold saline, and stored at − 80 °C. Tissue samples were used to investigate the effects of the studied drugs on tissue expression of TNF-α, using ELISA technique, NF-κB-p65 and RANKL by Western blotting with the V3 Western Workflow™ Complete System (Bio-Rad, Hercules, CA, USA), and MMP-9 via qRT-PCR. The assays were performed following the instructions provided with the commercial kits.

### Colon tissue parameters

#### Assessment of oxidative stress biomarkers

Colon segments were excised and analysed for oxidative stress and antioxidant status by measuring myeloperoxidase (MPO) activity, reduced glutathione (GSH), and malondialdehyde (MDA). MPO activity was determined using a modified version of (Krawisz et al. 1984) biochemical assay method. The reduced GSH level was calculated after measurement of the total GSH and GSSG enzymatic activity by the method described by (Griffith 1980). A colorimetric technique utilizing the thiobarbituric acid (TBA) determination method was utilized for measuring the MDA content (Draper et al. 1990).

#### Assessment of pro-inflammatory biomarkers

After animal sacrifice, colon segments were preserved in − 80 °C for later use in the evaluation of the tissue expression levels of TNF-α, using ELISA technique, NF-κB-p65 and MMP-9 by Western blot technique, and ICAM-1 using qRT-PCR. The essays were performed following the instructions provided with the commercial kits.

### Western blot analysis

For western blot analysis, tibiotarsal joint and colon tissue samples were collected and immediately stored at − 80 °C until use. Tissues were homogenized in ice-cold RIPA buffer supplemented with protease and phosphatase inhibitors. The homogenates were centrifuged at 12,000 × *g* for 15 min at 4 °C, and the resulting supernatants were collected for protein quantification using the Bradford assay. Equal amounts of protein were separated by SDS-PAGE using a Bio-Rad Mini-Protein II system and transferred to polyvinylidene difluoride membranes (PVDF) (Pierce, Rockford, IL, USA) with a Bio- Rad Trans-Blot system. Membranes were blocked with 5% non-fat dry milk in TBST and incubated overnight at 4 °C with primary antibodies specific to NF-κB-p65, RANKL, and MMP-9. After washing, membranes were incubated with appropriate HRP-conjugated secondary antibodies. Protein bands were visualized using an enhanced chemiluminescence (ECL) detection system and quantified by densitometry. β-actin was used as the loading control.

### Quantitative reverse transcription polymerase chain reaction (qRT-PCR)

Total RNAs were isolated from the homogenized joint and colon tissues using QIAzol Lysis Reagent and miRNeasy Mini Kit (Qiagen, Germany). cDNA was obtained by reverse transcription using the Maxime™ RT PreMix kit (Oligo dT15 Primer) (iNtRON Biotechnology Inc, Korea). Quantitative PCR was applied to determine the relative expression of joint MMP-9 and colon ICAM-1 genes using the specific primer sets for each gene in Table 1. The relative transcription levels were calculated using the ΔΔCt method by (Livak and Schmittgen 2001).

**Table 1:** Primer sequences

Gene	Forward & reverse primer sequence	
MMP-9	F	5′-TGCTGCCTATGAGGCTCACAAC-3′
	R	5′-GGAGGAAAACCGAGAGTGTGGA-3′
ICAM-1	F	5′-GGGCCCCCTACCTTAGGAA-3′
	R	5′-GGGACAGTGTCCCAGCTTTC-3′

### Histopathological examination

On day 29, hind paws and colon segments of rats were removed and processed for histopathological examination. Tissue samples were immediately fixed in 10% neutral-buffered formalin for 24–48 h. Following fixation, samples were dehydrated and embedded in paraffin blocks. Serial Sects.  (4–5 μm thick) were cut using a microtome and mounted on glass slides. Sections were stained with hematoxylin and eosin (H&E) for general histological evaluation of inflammatory cell infiltration, tissue architecture, and pathological alterations.

### Statistical analysis

Statistical analysis of data was performed using GraphPad Prism v7.0 (GraphPad Prism Inc., La Jolla, CA, USA). Mean ± SD was used to characterize quantitative data. One-way ANOVA and Tukey’s post hoc test for pairwise comparison were used for multiple comparisons of normally distributed variables. ANOVA was employed for scoring and comparing more than two periods or stages. A *p*-value of less than 0.05 was considered statistically significant.

## Results

### Body weight changes

As shown in Table 2, the normal control rats’ body weight increased regularly throughout the study period, with a 19.8% increase from Day 0 to Day 29. On the other hand, the average BW of untreated AA rats was significantly reduced by 4.7% on the 29^th^ day of the study. All treated groups showed a significant increase in their BW as compared to the untreated AA group, with leflunomide treatment showing only a 1.6% increase in BW, followed by the combination group with a 7.93% increase, and lastly the ectoine group with the highest percentage in BW improvement, with a 12.9% at day 29.

**Table 2:** The effect of treatment of AA rats with Leflunomide (10 mg/kg/day, orally), Ectoine (100 mg/kg/day, orally), and their combination, for two weeks on the percentage change in body weight (BW) of rats assessed as the difference between day 0 and day 29 of the study period. Data presented as Mean ± SD of 8 rats.

Experimental groups	BW (g) on Day 0	BW (g) on Day 29	% Changes in BW
Normal	172 ± 2.5	206 ± 5.43	19.8% ± 3.04
Untreated AA	197 ± 4.47	187.8 ± 4.14	− 4.7% ± 0.9*
Leflunomide	196 ± 4.18	199.2 ± 5.26	1.6% ± 0.28*#
Ectoine	193 ± 4.47	218 ± 6.44	12.9% ± 2.55*#a
Combination	194 ± 4.41	209.4 ± 2.96	7.9% ±1.3*#ab

*P* < 0.05 as compared to (*) normal group, (#): untreated AA group, (a): Leflunomide-treated group, (b): Ectoine-treated group

### Spleen and liver mass indices

**Fig. 1:**
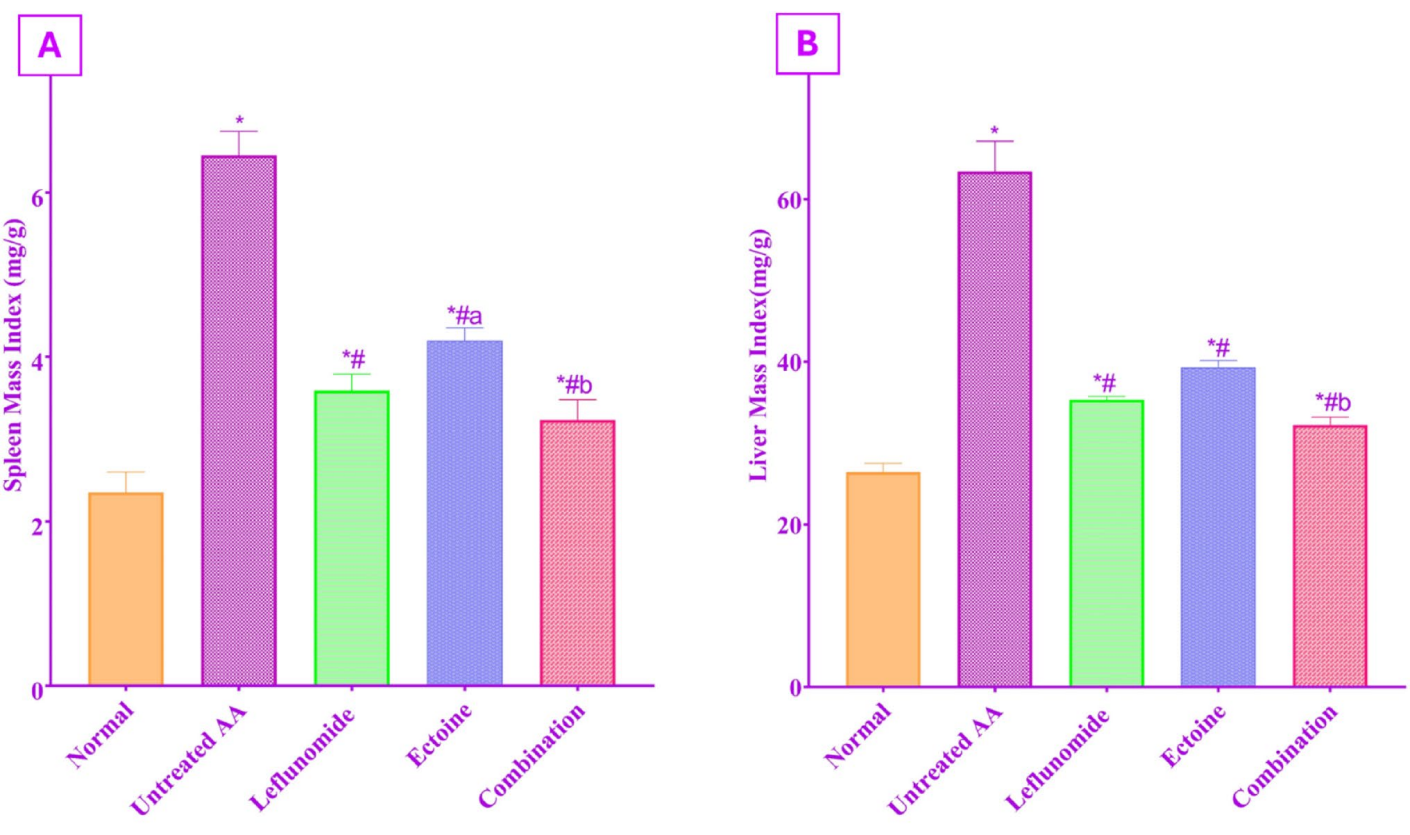
Change in the mass index of (**A**) Spleen, (**B**) liver following treatment with leflunomide (10 mg/kg/day, orally), ectoine (100 mg/kg/day, orally), and their combination, for 14 days, in adjuvant arthritic rats. Each value represents the mean ± SD of 8 rats, *P* < 0.05 as compared to: (*) Normal control group, (#) Untreated AA group, (a) Leflunomide-treated group, (b) Ectoine-treated group

As shown in Fig. 1, untreated AA rats exhibited a marked elevation in both spleen and liver mass indices compared with the normal control group (6.45 ± 0.29 vs. 2.35 ± 0.248 for the spleen; 63.46 ± 3.7 vs. 26.47 ± 1.06 for the liver; *P* < 0.001). Treatment with either leflunomide or ectoine significantly reduced spleen and liver mass indices relative to the untreated AA group, with no significant difference observed between these two treatment groups in liver mass index, while leflunomide caused a more significant reduction than ectoine in spleen mass index. Notably, the combination therapy produced the greatest reduction in both indices when compared with the untreated AA and ectoine groups, while showing no significant difference relative to the leflunomide-treated group.

### Hind paw swelling

**Fig. 2:**
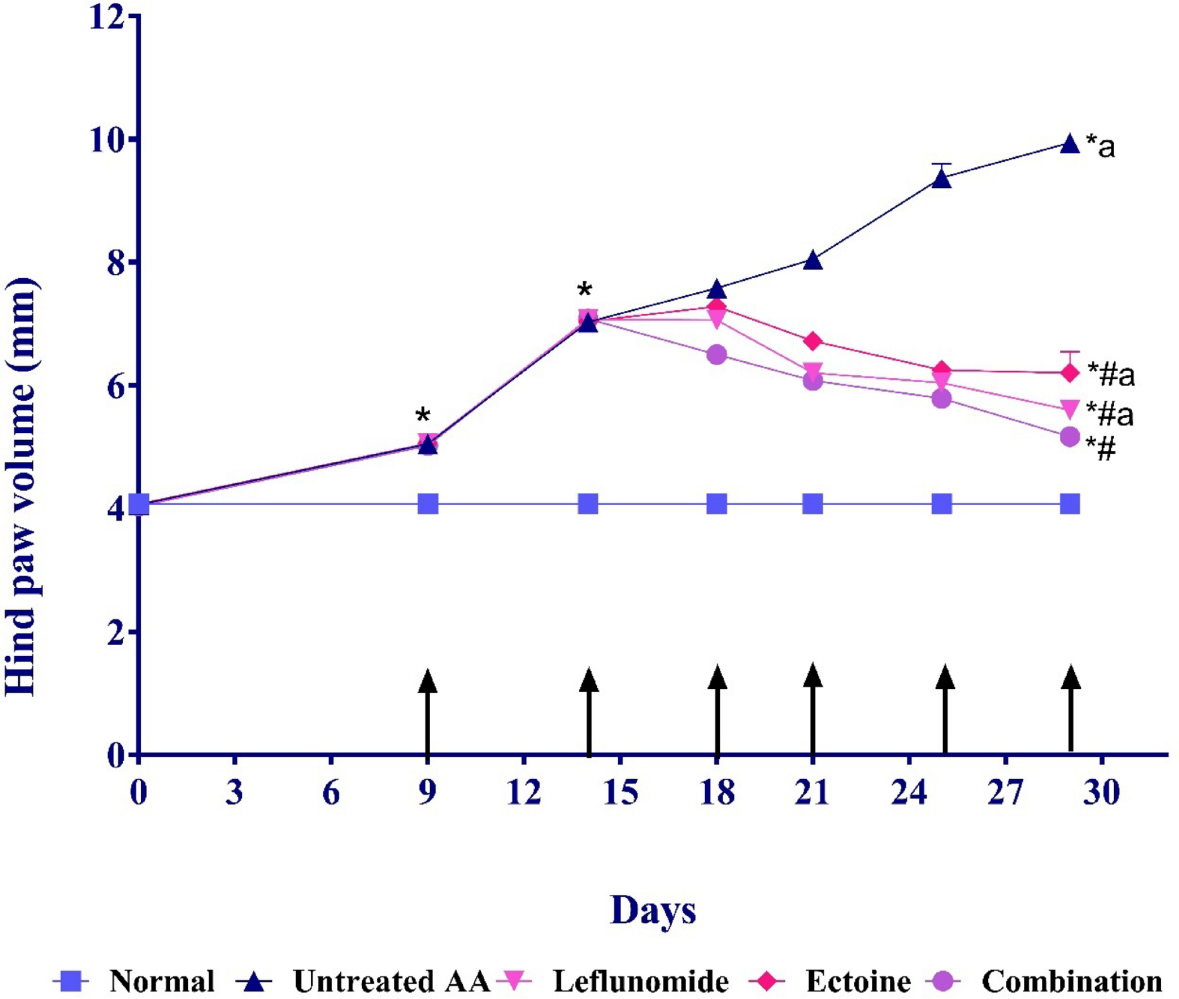
Evaluation of Hind Paw Swelling in AA rats on days 0, 9, 14, 18, 21, 25, and 29 following treatments with leflunomide (10 mg/kg/day, orally), ectoine (100 mg/kg/day, orally), and their combination, for two weeks. Data presented as the mean ± SD of 8 rats at each point with *P* < 0.001 as compared to (*) Normal control on days 9, 14, and 29, (#) Untreated AA group at day 29, (a) Combination Group at day 29

In the present study, the hind paw diameter was assessed at multiple times (days 0, 9, 14, 18, 21, 25, 29) as demonstrated in Fig. 2. All arthritic rats showed a progressive increase in hind paw swelling, reaching significantly high values on the 14^th^ day, post-adjuvant injection, as compared to day 0. Accordingly, treatment commenced on day 15 and continued for two weeks. In the untreated AA rats, hind paw swelling continued to increase progressively until the end of the treatment period, with a 2.44-fold increase observed on day 29. Different treatments resulted in gradual decreases in hind paw diameter compared to the untreated AIA group, with a significant reduction notably observed at the end of the treatment period. It is noteworthy that the combination group presented with the most significant retraction in hind paw swelling, with a 1.92-fold reduction as compared to untreated AIA on the 29th day of the experimental period.

### Serum inflammatory biomarkers

**Fig. 3:**
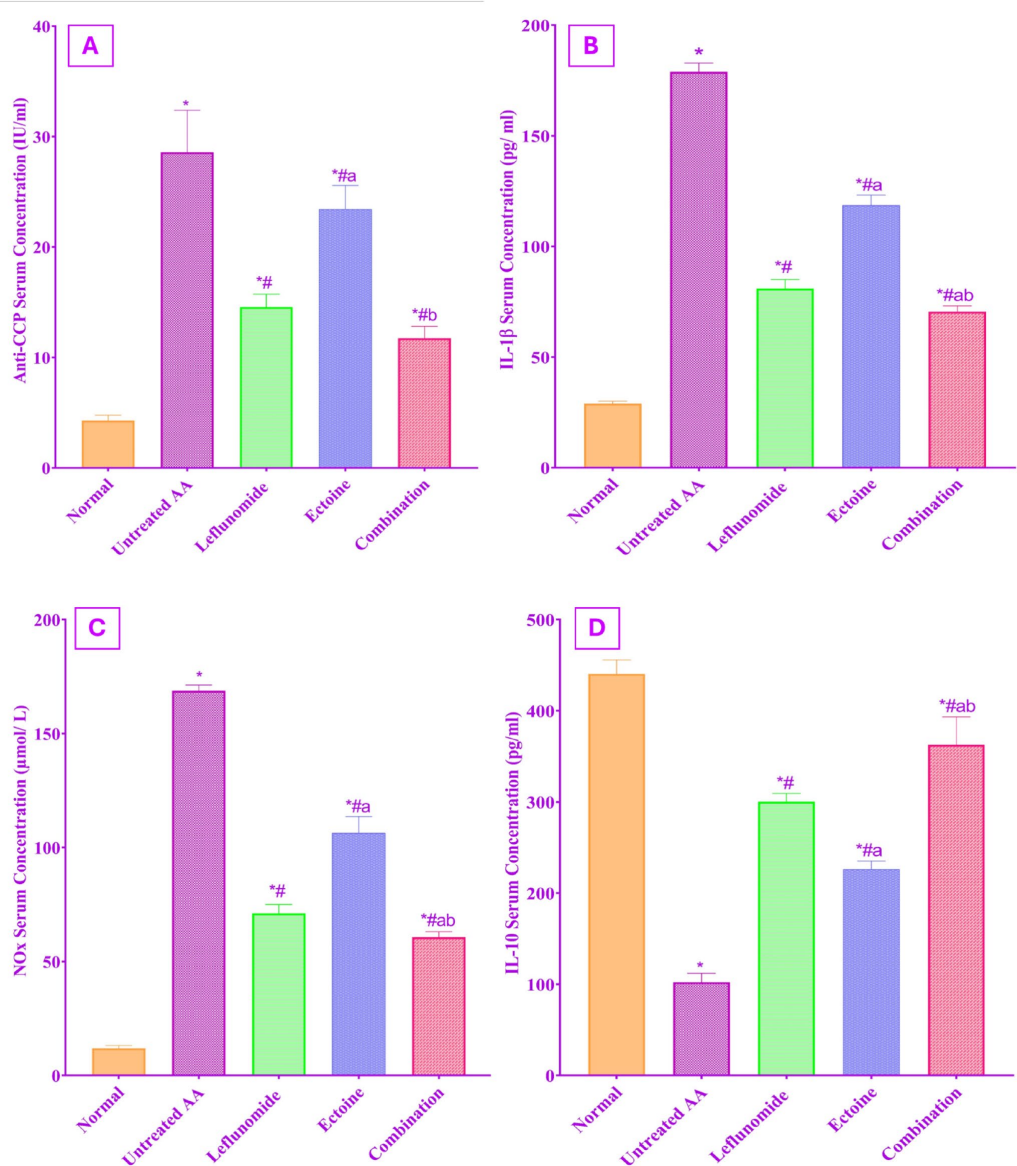
Evaluation of the serum concentration of: (**A**) Anti-CCP, (**B**) IL-1β, (**C**) NOx, (**D**) IL-10 following treatment with leflunomide (10 mg/kg/day, orally), ectoine (100 mg/kg/day, orally), and their combination, for 14 days, in adjuvant arthritic rats. Each value represents the mean ± SD of 8 rats, *P* < 0.05 as compared to: (*) Normal control group, (#) Untreated AA group, (a) Leflunomide-treated group, (b) Ectoine-treated group

The anti-inflammatory effect of both leflunomide and ectoine against AIA was evident in Fig. 3, which depicts changes in serum levels of Anti-CCP, IL-1β, IL-10, and NOx. There was a significant elevation in the pro-inflammatory parameters, Anti-CCP, IL-1β, and NOx, in the untreated AA group as compared to the normal control group.

All drug-treated groups showed a significant decrease in the serum Anti-CCP, IL1β, and NOx levels compared to the untreated AA group, with ectoine monotherapy exhibiting an inferior impact on Anti-CCP than leflunomide monotherapy. Meanwhile, leflunomide-treated rats showed significantly lower levels of both IL-1β and NOx as compared to ectoine-treated rats.

It is worth mentioning that the combination group offered the most significant effect among all treated groups on all pro-inflammatory parameters by reducing Anti-CCP by 58.96%, IL-1β by 60.58%, and NOx by 64.08% as compared to the untreated AA group.

On the other hand, Fig. 3D shows that the serum IL-10 level was significantly lower in untreated AA rats than in the normal control group. Treatment with leflunomide, ectoine, and a combination of both for 14 days resulted in a significant elevation in IL-10 concentration in comparison to the untreated AA group, with the most significant effect observed in the combination group that induced a 3.54-fold increase in IL-10 serum level as compared to the untreated AA group.

### Tibiotarsal tissue evaluation

#### Assessment of the expression levels of TNF-α and MMP-9

**Fig. 4:**
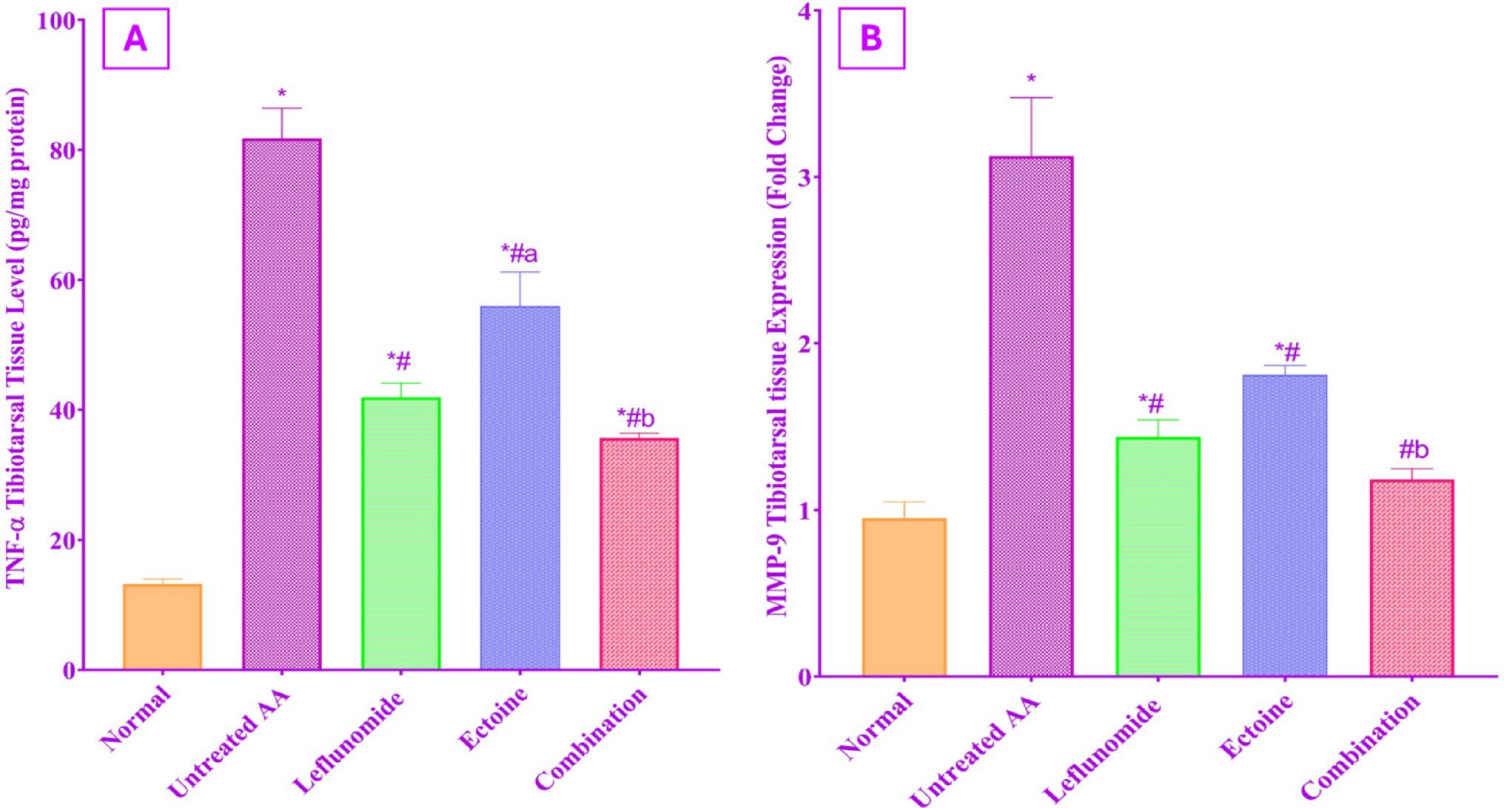
Determination of the effect of treatment with leflunomide, ectoine, and their combination, for 14 days, on (**A**) TNF-α and (**B**) MMP-9 in tibiotarsal tissue expression levels in AA rats. Each value represents the mean ± SD of 8 rats, *P* < 0.01 as compared to: (*) Normal control group, (#) Untreated AA group, (a) Leflunomide-treated group, (b) Ectoine-treated group

Figure 4 explains the changes in the tibiotarsal tissue expression levels of TNF-α and MMP-9, which were significantly increased in the untreated AA rats as compared to the normal control rats. Treatment of AA rats with leflunomide, ectoine, and their combination resulted in a significant suppression of TNF-α and MMP-9 expression, as compared to the untreated AA group. The impact of administration of either leflunomide or ectoine alone was comparable to each other on MMP-9 expression, while leflunomide showed superiority in TNF-α inhibition as compared to ectoine treated group.

The combination group significantly inhibited TNF-α tibiotarsal tissue expression more effectively than ectoine monotherapy, while its effect was not significantly different from leflunomide treatment. Meanwhile, combination therapy reduced the MMP-9 expression level to near normal levels with no significant difference from the negative control group.

#### Assessment of NF-κB-p65 and RANKL tissue expression

**Fig. 5:**
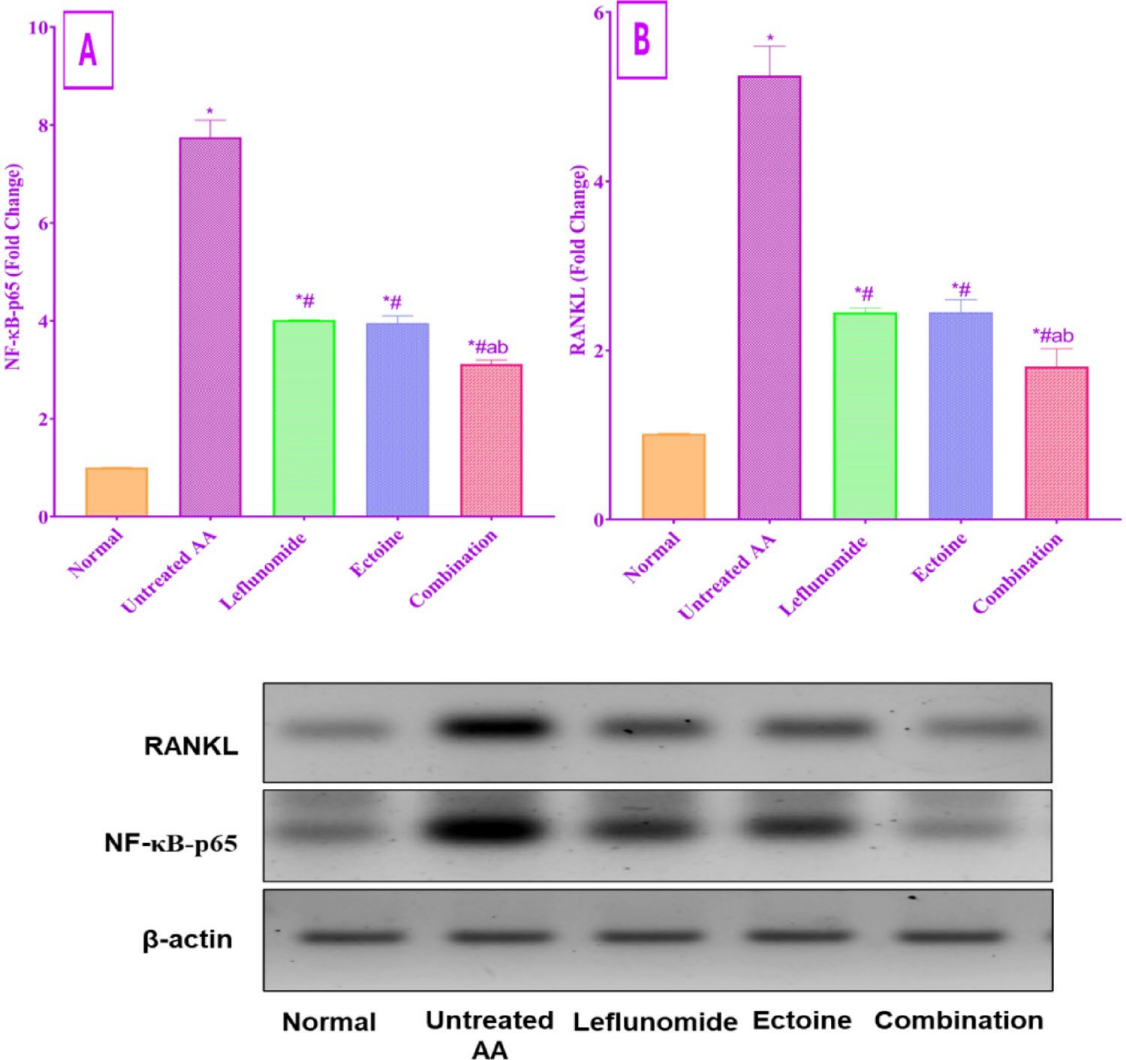
Evaluation of the effect of treatment with leflunomide, ectoine, and their combination, for 14 days, on (**A**) NF-κB-p65 and (**B**) RANKL tibiotarsal tissue expression in AA rats. Each value represents the mean ± SD of 8 rats, *P* < 0.01 as compared to: (*) Normal control group, (#) Untreated AA group, (a) Leflunomide-treated group, (b) Ectoine-treated group. The western blot images represent one rat per group

Induction of adjuvant arthritis resulted in a significantly enhanced tibiotarsal tissue expression of phosphorylated pro-inflammatory transcription factor NF-κB-p65 and its upstream activator RANKL, as observed in the untreated AA group. Administration of leflunomide, ectoine, or a combination of both for 14 days significantly inhibited the expression of both biomarkers compared to the untreated group, with the combination group offering the best results among all three groups, with a 59.74% reduction in NF-κB-p65 and 65.52% reduction in RANKL tissue expression when compared to the untreated AA group, as evident in Fig. 5.

### Colon tissue evaluation

#### Assessment of colonic tissue oxidative stress

**Fig. 6:**
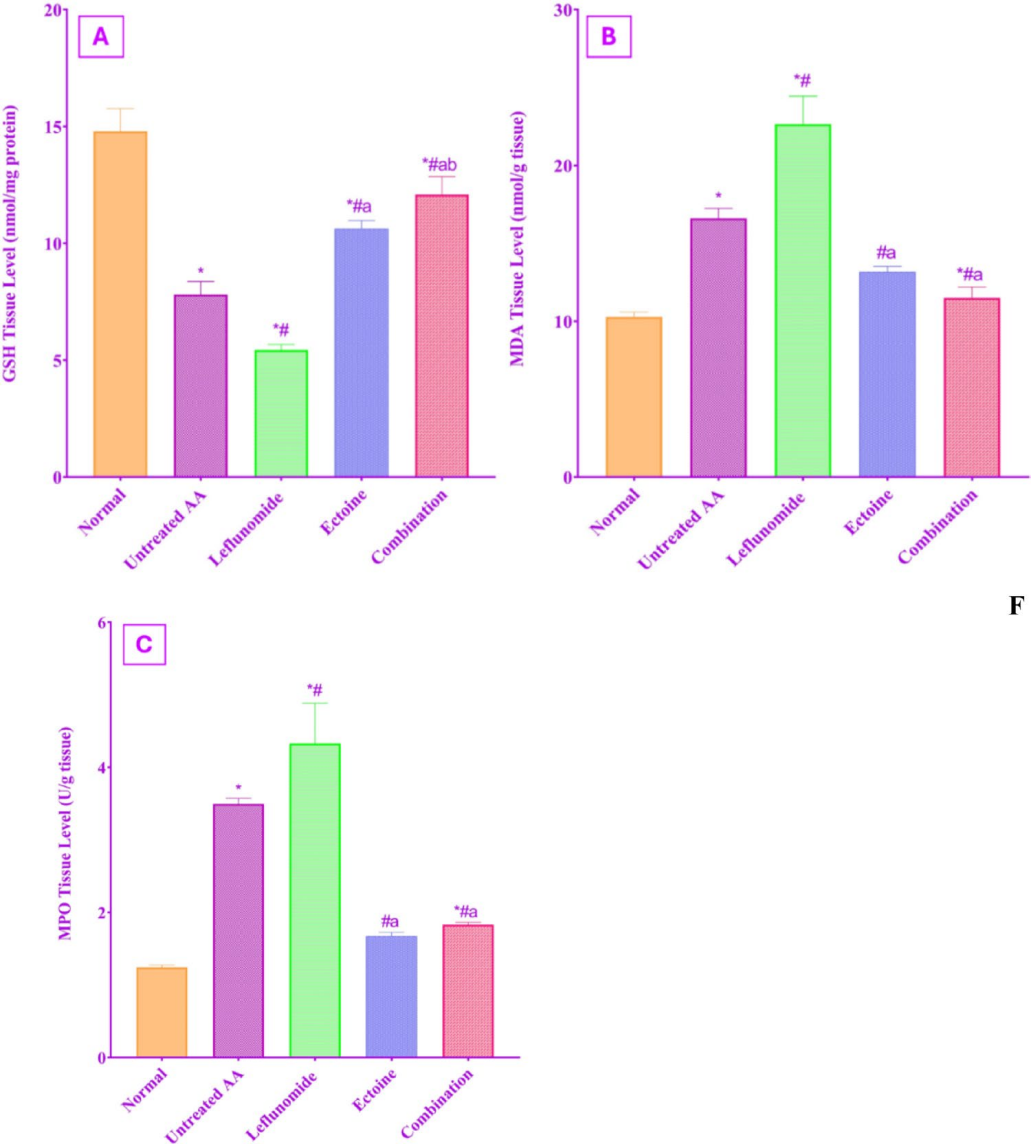
Effect of treatment with leflunomide, ectoine, and their combination, for 14 days, on (**A**) GSH, (**B**) MDA, and (**C**) MPO colonic tissue expression in AA rats. Each value represents the mean ± SD of 8 rats, *P* < 0.01 as compared to: (*) Normal control group, (#) Untreated AA group, (a) Leflunomide-treated group, (b) Ectoine-treated group

As demonstrated in Fig. 6, treatment with leflunomide was associated with a significant increase in oxidative stress burden on the colonic tissue, which is depicted as a significant increase in both MDA and MPO activity, together with a significant depletion of colonic GSH content as compared to normal control rats. Despite that, untreated AA rats showed a significant increase in oxidative stress when compared to negative control rats or to any of the treated groups; unfortunately, leflunomide therapy caused the most significant increase in oxidative stress relative to the untreated AA group. On the other hand, ectoine-treated rats presented with a significant reduction in both MDA and MPO to normal levels and a significant replenishment of GSH concentration to near normal levels as compared to negative control rats. The antioxidant effect of ectoine was significantly salient with a 2.22-fold increase in GSH expression, a 1.96-fold and a 2.57-fold reduction in MDA and MPO, respectively, as compared to the leflunomide group. Combining ectoine with leflunomide significantly ameliorated the oxidative imbalance with a 1.96-fold increase in GSH and 1.71-fold and 2.34-fold reduction in MDA and MPO, respectively, as compared to administration of leflunomide monotherapy. There was no significant difference between the impact of using ectoine alone or in combination regarding the activity of MDA and MPO, while ectoine monotherapy was more significantly efficient in preserving the GSH expression than combination therapy.

#### Assessment of TNF-α/NF-κB-p65/ICAM-1 and MMP-9 expression

**Fig. 7:**
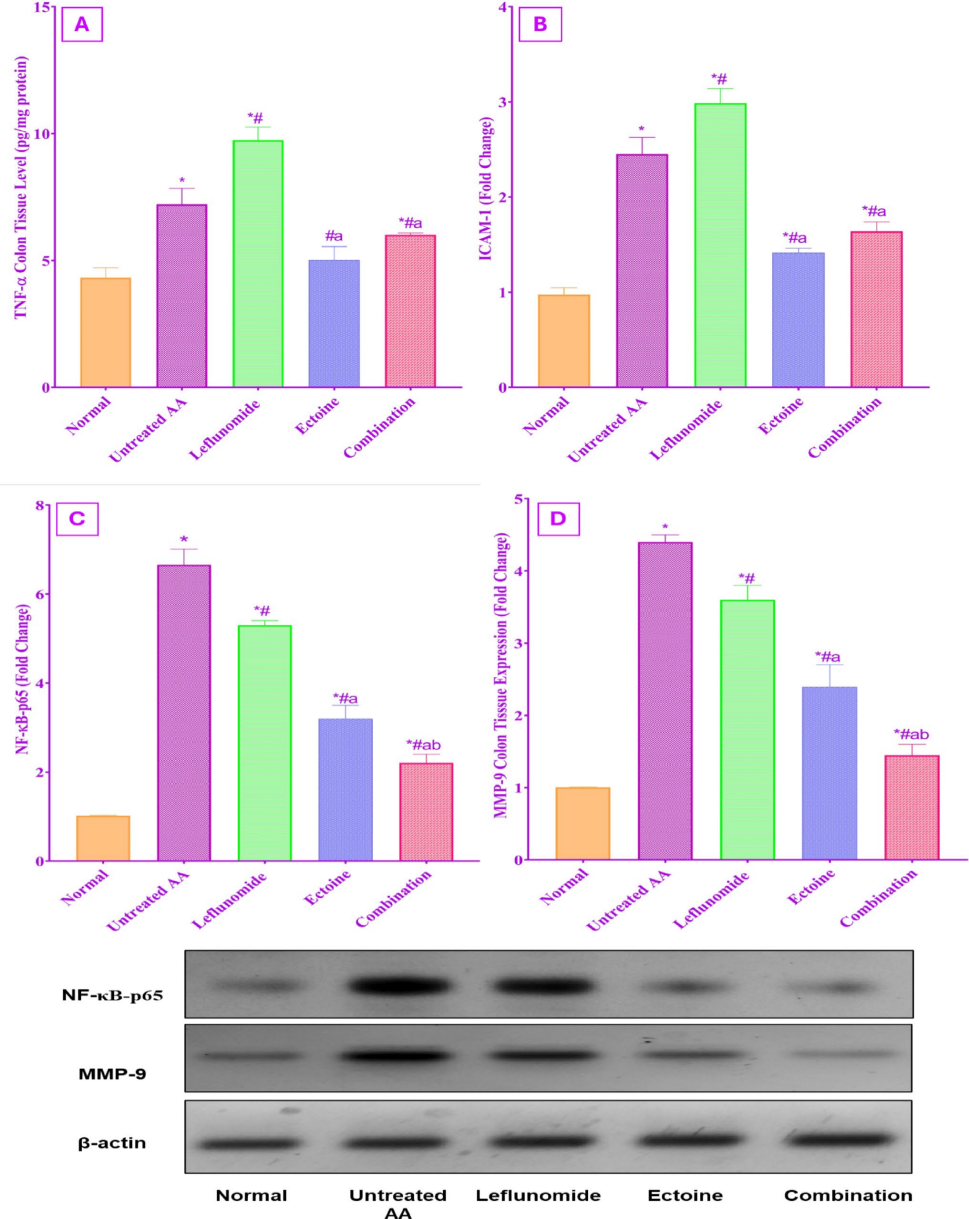
Determination of the effect of treatment with leflunomide, ectoine, and their combination, for 14 days, on (**A**) TNF-α, (**B**) ICAM-1, (**C**) NF-κB, and (**D**) MMP-9 colonic tissue expression in AA rats. Data represent the mean ± SD of 8 rats, *P* < 0.01 as compared to: (*) Normal control group, (#) Untreated AA group, (a) Leflunomide-treated group, (b) Ectoine-treated group. The western blot images represent one rat per group

Despite the significant increase in both TNF-α and ICAM-1 colon tissue expression among the untreated AA rats as compared to the control rats, leflunomide-treated rats exhibited a more significant rise in TNF-α and ICAM-1 tissue levels as compared to either untreated AA rats or any other treated group. Meanwhile, ectoine therapy significantly succeeded in diminishing TNF-α expression to normal levels and ICAM-1 to near normal levels with a 1.47-fold and 2.11-fold reduction in TNF-α and ICAM-1 colonic expression as compared to leflunomide therapy. Combination therapy showed a comparable activity to ectoine monotherapy in repressing TNF-α by 1.46-fold and ICAM-1 by 1.82-fold from the levels reached in leflunomide-treated rats, as depicted in Fig. 7A and B.

NF-κB-p65 and MMP-9 colon tissue expression were significantly increased in the untreated AA group by 6.05-fold and 4.37-fold, respectively, as compared to the control group. A significantly enhanced expression of both proteins was present in the leflunomide-treated group as compared to the ectoine or combination groups. Ectoine administration mended the inflammatory process by significantly reducing the expression of NF-κB-p65 by 2.08-fold as compared to the untreated group and by 1.65-fold as compared to leflunomide treatment. Moreover, ectoine restrained MMP-9 tissue expression and decreased it by 1.84-fold as compared to the untreated AA group and 1.5-fold as compared to the leflunomide group. Furthermore, Fig. 7C and D illustrate that the combination of both ectoine and leflunomide displayed the most significant decrease in both NF-κB-p65 and MMP-9 colonic expression among all treated groups as compared to either the untreated AA group, ectoine group, or leflunomide group.

### Histopathological examination

#### Hind paws

**Fig. 8:**
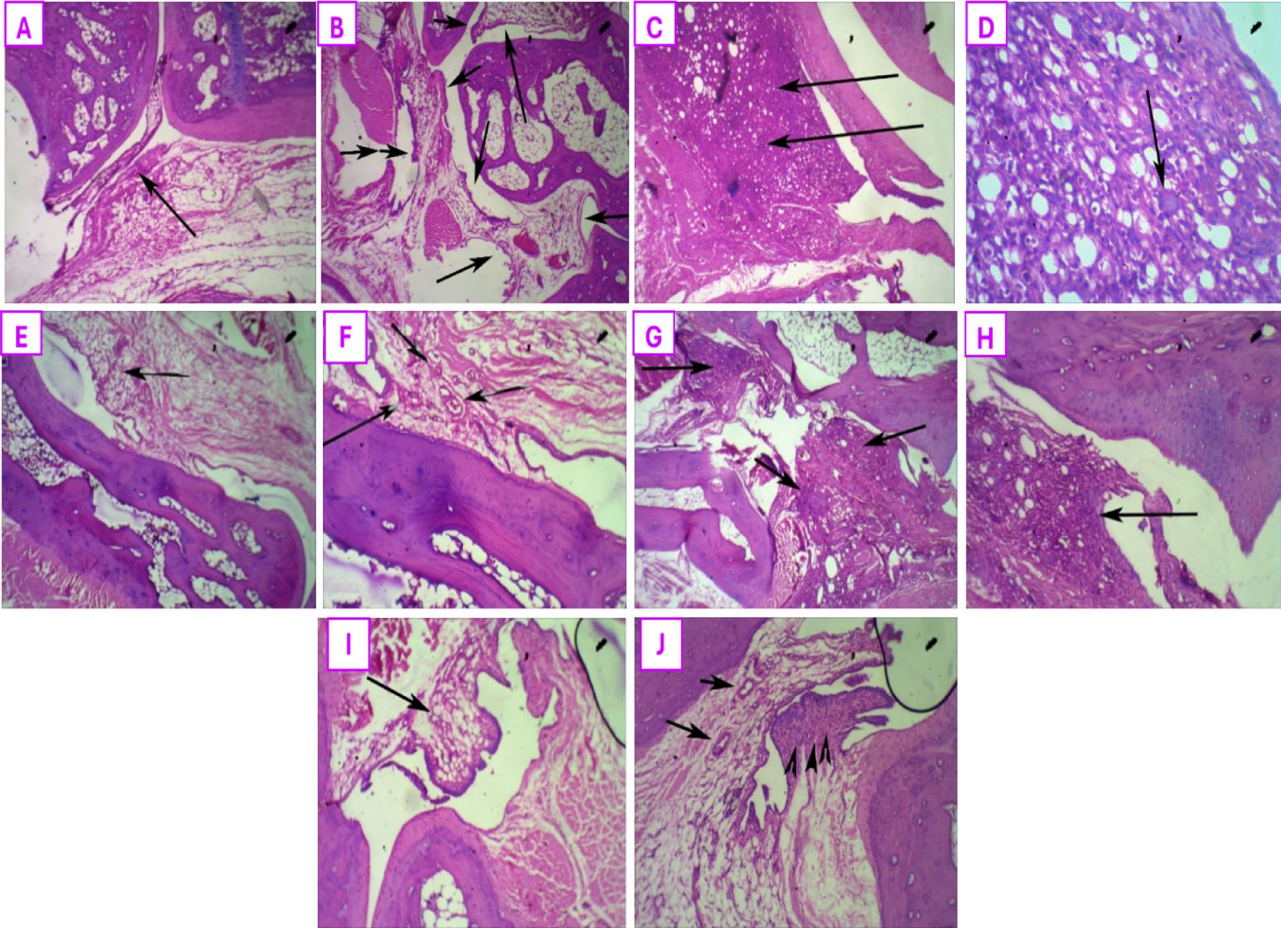
Effect of treatment with leflunomide, ectoine, and their combination, for 14 days, on the histopathological changes in AA rats’ hind paw sections: (**A**) Hind paw section of a normal control rat showing intact architecture with no evidence of inflammation (black arrow), (**B**) Hind paw section of a normal control rat showing normal synovium with absence of inflammation (Black arrows) (**C**) Hind paw section of untreated AA showing severe synovial inflammation (black arrows), (**D**) Hind paw section of untreated AA showing severe inflammatory infiltrate in synovium and a giant cell (black arrow), (**E**) Hind paw section of leflunomide-treated rat showing near normal joint morphology and minimal inflammation (black arrow). (**F**) Hind paw section of leflunomide-treated rat showing mild congestive arthritis (black arrows pointing at the congested vessels), (**G**) Ectoine-treated hind paw section showing congested synovium with mild inflammation (black arrows), (**H**) Ectoine-treated hind paw section showing moderate synovial inflammation (black arrow), (**I**) Hind paw section of combination therapy group showing near normal joint architecture and no inflammation (black arrow), and (**J**) Hind paw section of combination therapy group showing mild synovial congestion (black arrows) with absence of inflammation (arrowheads).

Histopathological examination of the hind paws of rats was done to display the major pathological findings using H&E stain, as demonstrated in Fig. 8. The normal control rats presented with a normal synovial architecture, normal joint spaces with no sign of inflammation as depicted in Fig. 8A and B. In contrast, the untreated AA rats’ hind paws showed dense inflammatory infiltration around the synovial lining with the presence of giant cells, as observed in Fig. 8C and D. These changes were remarkably improved in all treated groups where leflunomide therapy managed to preserve a near normal joint morphology with minimal inflammation, Fig. 8E, and mild congestive arthritic changes as seen in Fig. 8F. Ectoine-treated rats showed congested synovium with mild inflammation in Fig. 8G and H, while the combination of both therapies led to an evident improvement in arthritic signs with a near normal architecture of the joint with the absence of inflammation as depicted in Fig. 8I and J.

#### Colon

**Fig. 9:**
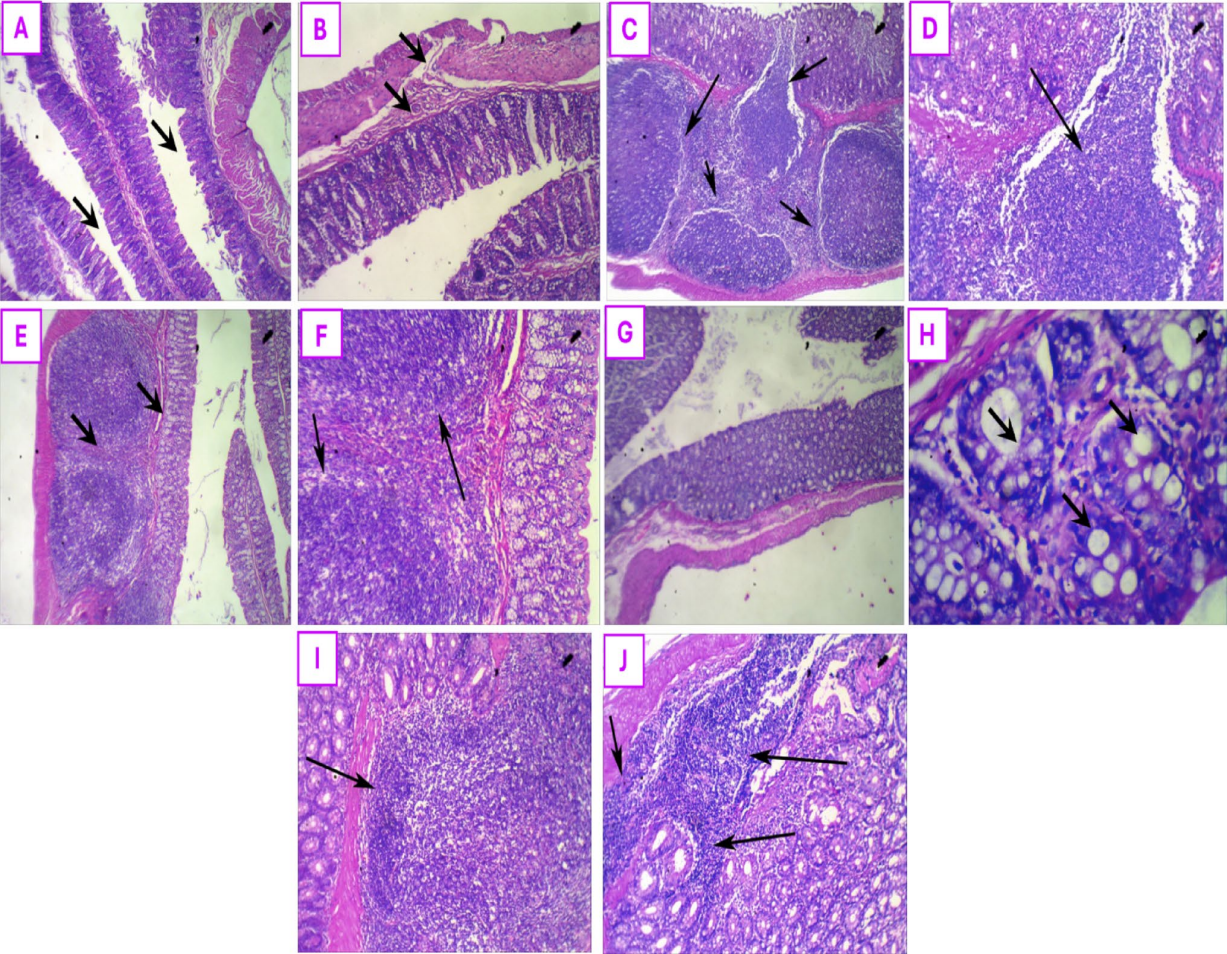
Effect of treatment with leflunomide, ectoine, and their combination, for 14 days, on the histopathological changes in AIA rats’ colon sections: (**A**) Colon sections from normal control group with normal morphology and no inflammation (black arrows), (**B**) Colon section from normal colon showing mucosa and muscularis with preserved architecture (black arrows), (**C**) Section of colon from untreated AA rat showing heavy transmural lymphoid infiltrate with large reactive lymphoid follicles (black arrows), (**D**) Section of colon from untreated AA rat showing lymphoid inflammatory infiltrate crossing the muscularis mucosa (black arrow), (**E**) Colon section from leflunomide-treated rat showing heavy lymphoid infiltrate in colonic wall involving mucosa and submucosa (black arrows), (**F**) Colon section from leflunomide-treated rat showing large submucosal lymphoid follicles (Black arrows), (**G**) Colon section from ectoine-treated rat showing near normal morphology, (**H**) Colon section from ectoine-treated rat showing mild inflammatory infiltrate in the lamina propria with near normal morphology and intact muscularis (black arrows), (**I**) Section of colon from the combination therapy-treated group showing dense submucosal reactive lymphoid follicles (black arrow), and (**J**) Section of colon from combination therapy-treated group showing moderate mucosal and submucosal inflammatory infiltrate (Black arrows)

Histopathological examination of colon sections was performed using H&E stain to evaluate the impact of disease induction as well as different therapeutic options on the colonic integrity. As depicted in Fig. 9A and B, the normal control rats showed a preserved architecture of colonic mucosa and muscularis. Meanwhile, colon sections obtained from the untreated AA rats exhibited heavy transmural lymphoid infiltration with large reactive lymphoid follicles, Fig. 9C, in addition to lymphoid inflammatory infiltrate crossing the muscularis mucosa, Fig. 9D. Leflunomide administration for 14 days caused heavy lymphoid infiltration in the colonic wall involving mucosa and submucosa, Fig. 9E, with large submucosal lymphoid follicles as shown in Fig. 9F. On the other hand, ectoine-monotherapy showed and efficient improvement with a near normal colonic morphology, Fig. 9G, with mild inflammatory infiltration in the lamina propria and near normal morphology with intact muscularis as seen in Fig. 9H. The colonic tissue sections obtained from rats receiving a combination therapy presented with a better colonic morphology than the leflunomide-treated group, with dense submucosal reactive lymphoid follicles, Fig. 9I, and moderate mucosal & submucosal inflammatory infiltration, Fig. 9J.

## Discussion

Rheumatoid arthritis is the most prevalent autoimmune inflammatory arthritis disorder among adults. Although there are multiple lines of therapy, it continues to be an incurable disorder with an intricate pathophysiology that needs further exploration (Jain et al. 2025). One of the most crucial DMARDs that has long been employed in the management of RA-refractory cases is Leflunomide (Behrens et al. 2021). Regrettably, the utilization of leflunomide is frequently associated with numerous side effects, such as diarrhea, colitis, and hepatotoxicity, that may necessitate the discontinuation of therapy (Narváez et al. 2015). The diverse adverse effects of conventional and biological agents used for the treatment of RA fostered the search for novel agents that provide adequate disease control with minimal potential for long-term side effects.

In the current study, we employed an AIA model in rats, as it is one of the most reliable models resembling the hallmarks encountered in human RA. (Bevaart et al. 2010). In line with previous studies, a progressive increase in the width of the tibiotarsal joints was significantly noted in the untreated AA rats, as an indicator of arthritis severity (Yang et al. 2019).Consistent with prior findings, leflunomide significantly decreased hind paw edema and inflammation, likely by disrupting the cell cycle with a subsequent inhibition of the production of pro-inflammatory mediators (Gowayed et al. 2015). Our work highlighted the anti-inflammatory effect of ectoine, evident by the significant reduction in the hind paw swelling and the amelioration in the inflammatory process in the affected joints. Despite that, leflunomide was superior to ectoine monotherapy; the combination therapy offered the most significant retraction in hind paw swelling and arthritis progression. The precise molecular mechanism behind the protective action of ectoine remains inadequately elucidated; nonetheless, it is posited to stem from its capacity to diminish ceramide formation, which is regarded as a mediator of inflammation (Bethlehem and Echten-Deckert 2021).

In the untreated arthritic group, liver and spleen mass indices were significantly increased, as compared to the normal control group. A similar effect was reported in collagen induced arthritis (CIA) model, with a close correlation to inflammatory propagation (Lee and Im 2025). These effects were significantly restored by ectoine administration. The beneficial effect of leflunomide on spleen and liver mass indices was supported by similar findings in adjuvant arthritis rats (Gowayed et al. 2015). Similarly, ectoine was reported to nearly normalize spleen mass index in an experimental model of colitis in rats, because of its anti-inflammatory effect (Abdel-Aziz et al. 2013). The reduction in both organ mass indices was more profound in the combined therapy group.

Anti-CCP antibodies are implicated in the pathogenesis of RA mainly by mediating osteoclastogenesis and bone erosion (Kurowska et al. 2017). In the present study, serum levels of anti-CCP antibodies were significantly elevated in the untreated AA group, compared to the normal control group, as observed earlier (Corrales et al. 2019). The significant downregulation of Anti-CCP by leflunomide treatment observed in our study was previously reported in AIA (Behrens et al. 2021). Treatment with ectoine alone significantly lowered Anti-CCP levels, as compared to the untreated group. Its combination with leflunomide had a stronger impact, causing a more prominent decline in the Anti-CCP levels than leflunomide alone. Such effect is supported by previous findings of better response of Anti-CCP positive patients, to a combination of other DMARDs or natural extracts with leflunomide (Xiang et al. 2015).

The present findings demonstrate a significant elevation of serum nitric oxide (NOx) levels in untreated AA rats, reflecting the role of NOx in inducing T-cell dysfunction and creating an oxidative stress milieu, in addition to expanding the generation of pro-inflammatory cytokines (Huang et al. 2023). Conversely, the significant reduction in serum NOx levels observed following leflunomide treatment may be attributed to a direct inhibitory effect on inducible nitric oxide synthase (iNOS) (Hadi et al. 2022). In the present study, ectoine treatment significantly reduced elevated serum NOx levels in AA rats, consistent with previous findings in an ischemia–reperfusion model, where ectoine decreased serum NOx and conferred intestinal protection (Pech et al. 2012). This effect may be attributed to attenuation of oxidative stress and inhibition of pro-inflammatory mediators, including IL-1β, TNF-α, and NF-κB, which regulate iNOS expression (Huang et al. 2023).

TNF-α and IL-1β are pivotal drivers of the inflammatory cascade responsible for sustaining joint inflammation and cartilage destruction (Zhang et al. 2021). In contrast, IL-10 is a potent immunomodulatory cytokine that attenuates inflammation and bone erosion by preserving joint integrity and suppressing TNF-α and IL-1β production (Ge et al. 2020).

According to our findings, serum IL-1β and joint TNF-α expression levels were significantly elevated in the untreated AA group, together with a significant decrease in serum IL-10 concentration. These findings agree with recent studies in a similar animal model (Waseem et al. 2025). Meanwhile, leflunomide dampened the upsurge in inflammatory cytokines together with a significant enhancement of IL-10 production as reported earlier (Abbas et al. 2022; Gowayed et al. 2015). Our work elucidated that ectoine possesses a significant anti-inflammatory activity presented by its significant prohibition on IL-1β and TNF-α expression, with a significantly opposing action on IL-10. Again, the favourable anti-inflammatory effect of ectoine in combination with leflunomide was significantly eminent to either drug alone. Earlier investigations attribute the impact of ectoine on pro-inflammatory cytokines to its action as a bio-membrane stabilizer, causing high membrane fluidity and conferring resilience against infiltration by inflammatory triggers (Bethlehem and Echten-Deckert 2021; Merckx et al. 2022).

In RA, the transcription factor, NF-κB, constitutes a central inflammatory signalling pathway driving the expression of genes involved in the inflammatory response (Giridharan and Srinivasan 2018). There is a bidirectional relationship between pro-inflammatory cytokines IL-1β and TNF-α with NF-κB-p65, where they promote the expression of each other in a vicious cycle that ultimately results in fibroblast-like synoviocytes (FLS) proliferation and invasiveness that promote the major pathological features in RA (Li et al. 2020).

The findings of our investigations showed a significant increase in NF-κB-p65 tibiotarsal tissue expression in the untreated AA group, while leflunomide therapy significantly abrogated its articular tissue expression, an effect that was previously suggested to be through inhibition of TNF-α-induced activation of NF-κB-p65, suppression of IKβ degradation, and blockade of NF-κB receptor gene expression (Cutolo et al. 2006). Ectoine administration presented a comparable inhibition of NF-κB-p65 tibiotarsal tissue expression to leflunomide therapy, while using both drugs together induced the most significant impact, emphasizing the leverage of combination therapy on combating the severity and progression of inflammation. Our results were supported by previous reports of the ability of ectoine to reduce the translocation of NF-kβ, which prevents the subsequent activation of inflammatory cascades and reduces the expression TNF-α, IL-1β, IL-6, and COX-2 (Bethlehem and Echten-Deckert 2021).RANKL promotes osteoclastogenesis and systemic inflammation, dominating the pathophysiology of RA (Papadaki et al. 2019). TNF-α and IL-1β are the primary triggers that enhance RANKL expression in osteoclasts and promote bone resorption (Liu et al. 2017). Furthermore, these pro-inflammatory cytokines stimulate the transcription of matrix metalloproteinases, and on top of them, MMP-9 via activation of MAPK/ NF-κB and AP-1, thereby amplifying inflammation and extracellular matrix (ECM) degradation (Araki and Mimura 2017).

In the present study, RANKL and MMP-9 tibiotarsal tissue expression were evidently increased in the untreated AA group. These findings are in strong consonance with earlier studies in arthritic rats (Stojanovic et al. 2023; Zhao, Z. et al., 2021). Meanwhile, our work demonstrated ectoine repressive action on RANKL and MMP-9 expression to a level comparable to leflunomide therapy. The combination of both drugs elicited a greater inhibitory effect than either drug alone, with a near normal MMP-9, which is supported by recent evidence showing that ectoine downregulated MMP-9 expression in chondrocytes in an in vitro osteoarthritis investigation (Li et al. 2024). While the observed RANKL tissue in ectoine-treated rats can be attributed to the remarkable inhibition of other pro-inflammatory mediators dominating the NF-kβ-p65 signaling pathway (Zhao et al. 2021a, b).

In the current work, histopathological examination of the hind paws of rats revealed a significant synovial tissue inflammatory infiltration in the untreated AA group, which signifies the AIA characteristic tissue damage as reported previously (El-Sayyad et al. 2021). While leflunomide therapy resulted in remarkable preservation of near-normal joint structure with minimal inflammation. Similarly, treatment with ectoine resulted in mild synovial congestion, with the combination group reverting the synovial structure to the near normal form. This comes to affirm the anti-arthritic potential of ectoine and reflects the above-mentioned anti-inflammatory effect on articular tissue.

On another aspect, the histopathological examination of colon segments of untreated AA rats in the current study showed transmural lymphoid inflammatory infiltration crossing the muscularis mucosa, confirming previous reports implicating intestinal inflammation with progression of RA, because of abnormalities in the intestinal mucosal immunity by an imbalance of Th17/Treg cells in colon tissue (Qin et al. 2023). In contrast to leflunomide’s prominent articular preserving action demonstrated in the present work, heavier lymphoid infiltration of the colonic wall, with large submucosal lymphoid follicles were found after its administration to AA rats. A causal relationship between leflunomide and colitis was concluded before, where similar lymphocytic colitis was reported upon histopathological examination of colon biopsies obtained from rheumatoid arthritis patients treated with leflunomide (Gugenberger et al. 2008; Kwok and Morosin 2019).

On the other hand, ectoine monotherapy ameliorated intestinal inflammation, caused by AA induction, with only mild inflammatory infiltration left in the colonic mucosa. Colon tissues from the combination group showed moderate mucosal and submucosal inflammatory reactions. These results could be greatly due to ectoine’s intestinal membrane-stabilizing effect, since recent evidence stated that the disruption of intestinal barrier integrity has been demonstrated to precede the inflammatory stage of collagen-induced arthritis (CIA), in mice and human arthritis (Heidt et al. 2023; Simpkins et al. 2022).

Rats in the untreated AA group demonstrated significant weight loss in the present study, which has been previously explained as a consequence of the systemic inflammatory process (Wang et al. 2016). The previously discussed articular anti-inflammatory effects of leflunomide could have prevented body weight loss in the leflunomide-treated rats. However, rats showed a significantly lower change in BW by the end of the study as compared to other treated groups. This can be closely related to the histopathological evidence of colonic injury demonstrated in our findings. Treatment with ectoine demonstrated the most significant improvement in body weight, followed by the combination group in second place. These findings can be explained by the evident intestinal protective effect of ectoine, documented in the histopathological examination and attributed to the anti-inflammatory and mucosal preservative impact previously reported (Abdel-Aziz et al. 2013).

Further analysis of the colon tissues was performed to define the mechanisms involved in the observed protective effects of ectoine. High MPO activity was correlated to neutrophilic infiltration into the colon, and to overproduction of reactive oxygen species in colon tissues (Baum et al. 2025). This comes in agreement with our findings, where MPO activity was significantly increased in untreated AA, in association with an increase in MDA concentration and depletion of GSH content in the colonic tissue. While leflunomide treatment exposed the colon tissue to the most significant oxidative stress-induced damage, by showing a higher MPO, MDA levels, and significantly lower GSH concentration, which can explain the histopathological damage mentioned above.

Conversely, ectoine provided the most significant antioxidant capabilities by replenishing GSH stores in the intestinal tissue, with significant suppression of MPO activity and consequently the lipid peroxidation biomarker MDA to levels comparable to the normal control group. The combination of ectoine with leflunomide significantly prevented leflunomide-induced oxidative stress, with significant increases in GSH, together with MDA and MPO concentrations reduction. Such an antioxidant effect aligns with previous results of ectoine in experimental colitis in rats (Abdel-Aziz et al. 2013). Also, ectoine ROS scavenging ability was proven, and correlated to its anti-inflammatory effects observed in skin, lung, and IBD (Brands et al. 2019).

Inflammation and oxidative stress are extremely interlinked processes and are considered causes and consequences of cellular pathologies (Vona et al. 2021). All biomarkers of the inflammatory trajectory, TNF-α/ NF-β-p65/ ICAM-1, and MMP-9, were found to be abnormally expressed in the colon tissues of the untreated AA group, which is in accordance with previous evidence linking intestinal inflammation to the pathogenesis of rheumatoid arthritis (Tajik et al. 2020; Zaiss et al. 2021). The upregulation of this signaling pathway is accompanied by an MMP-9-induced increase in intestinal epithelial tight junction permeability (Al-Sadi et al. 2021) and facilitation of transmigration of leukocytes into the inflamed sites by increased ICAM- expression (Vestweber 2015).

Meanwhile, treatment of AA rats with leflunomide induced more deleterious effects on the studied colonic parameters, increasing colonic tissue expression of TNF-α/ NF-_k_β-p65/ ICAM-1, and MMP-9, in association with its induced raising of MPO, and MDA to a level higher than the untreated AA group and depletion of GSH. The leflunomide-induced colonic inflammatory damage was earlier documented in RA patients and resulted in poor compliance with treatment (Abbas et al. 2022; Kwok and Morosin 2019).

Besides combating colonic oxidative stress, the anti-inflammatory effect of ectoine, shown in the serum and the joints, extended to the colon, leading to significant reductions in TNF-α/ NF-κB-p65/ /ICAM-1 and MMP-9 tissue expression. Moreover, the anti-inflammatory effect of combination therapy was significant on all the studied parameters as compared to the leflunomide group. Similarly, ectoine showed a significant inhibitory effect on TNF-α /NF-_κ_β-p65/ ICAM-1 signaling and expression, with significant amelioration of pathological features of colitis in an experimental IBD model (Abdel-Aziz et al. 2013, 2015). Another justification for the anti-inflammatory properties of ectoine is via preservation of the integrity of the intestinal barriers, prohibiting cellular inflammatory translocation in the colonic submucosal (Vargas-Robles et al. 2019).

## Conclusions

The present study provides the first evidence that ectoine exerts anti-inflammatory effects in AIA. Ectoine significantly attenuated both articular and extraarticular inflammatory parameters, supporting its growing therapeutic potential in inflammatory disorders, particularly given its favorable safety and tolerability profile.

It abrogated AA-induced joint inflammation by suppressing serum IL-1β, Anti-CCP, and tissue TNF-α/ RANKL/ NF-κB-p5 and MMP-9 expression. Although its anti-rheumatic efficacy was less pronounced than that of leflunomide, combination therapy produced a superior influence in the restoration of inflammatory biomarkers to near-normal levels.

Ectoine presented a salvage against leflunomide-induced colonic inflammation by the inhibition of colonic inflammatory pathway TNF-α/ NF-κB-p65, ICAM-1/ MMP-9, most probably due to its membrane-stabilizing and antioxidant properties. These findings suggest that ectoine may serve as a valuable adjunct to leflunomide, enhancing therapeutic efficacy while limiting the frequently severe gastrointestinal adverse effects. Nevertheless, clinical studies are required to validate these protective effects. Furthermore, future investigations should explore whether early modulation of intestinal barrier integrity by ectoine can alter disease initiation or progression.

## Data Availability

No datasets were generated or analysed during the current study.
